# Development of a molecular method for the rapid screening and identification of the three functionally relevant polymorphisms in the human TAS2R38 receptor gene in studies of sensitivity to the bitter taste of PROP

**DOI:** 10.1186/s40064-015-1045-0

**Published:** 2015-06-09

**Authors:** Roberto Orrù, Elena Atzori, Alessandra Padiglia

**Affiliations:** Department of Life and Environment Sciences, University of Cagliari, 09042 Monserrato, CA Italy

**Keywords:** Bitter taste, TAS2R38, PROP taster status, PCR-RFLP, *CA6*

## Abstract

The objective of this work was to develop a rapid screening method to identify the three single nucleotide polymorphisms (SNPs) in the *TAS2R38* gene, with the aim of providing a significant contribution to studies designed to assess sensitivity to the bitter taste of 6-*n*-propylthiouracil (PROP). Specifically, the objective of this study was to characterize the *TAS2R38* gene haplotypes in a group of 60 subjects with variable sensitivity to PROP and preliminarily genotyped for the rs2274333 allele (A/G) of carbonic anhydrase isoform VI gene (*CA6*). The molecular characterization of the *TAS2R38* gene was conducted using the PCR-restriction fragment length polymorphism technique after creating artificial restriction sites upstream or downstream of the SNPs, as none of the three polymorphisms contributes to the formation of a restriction site for a specific endonuclease. The results indicate that the method described in this paper could be a valid and simple experimental strategy to identify genetic differences related to taste sensitivity to bitter taste, and could be applied as a nutrigenetics test in studies aimed at understanding people’s eating behaviors.

**Electronic supplementary material:**

The online version of this article (doi:10.1186/s40064-015-1045-0) contains supplementary material, which is available to authorized users.

## Background

**Figure 1:**
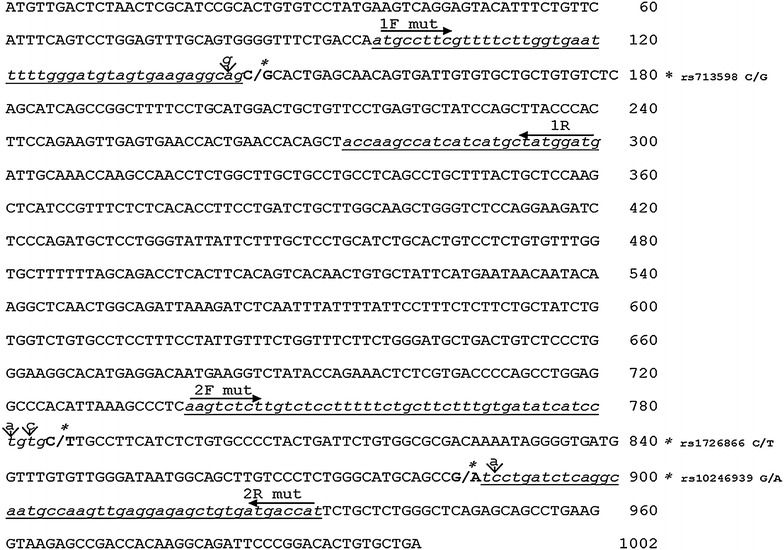
Nucleotide and deduced amino acid sequences of *TAS2R38* gene. The nucleotide sequence is numbered in the 5′–3′ direction. The primer sequences are in *lower-case letters* and *underlined*. *Arrows* above underlined sequences indicate sense (→) and antisense (←) primers used to amplify the genomic region covering the SNPs of interest (A/G), shown in *bold*, separated by a *forward slash*, and denoted with an *asterisk*. Nucleotides inserted into the primers to create mutagenesis in the fragment are shown above the sequence with an *arrow* (↓). The figure also shows the codes of the three SNPs.

The sensation of taste is the biochemical result of an intricate mechanism of signal transduction pathways, which begins at the level of the oral cavity and ends in the gustatory cortex of the primary central nervous system. Specifically, the path starts with the interaction of signaling molecules dissolved in saliva, with characteristic receptors located in the taste buds (Hamilton and Norgren 1984; Avenet and Lindemann 1989). Bitter taste is mediated by a family of about 30 different types of T2R receptors associated with G proteins (Margolskee 2001; Ueda et al. 2003), which bind about 500 different molecules, including divalent cations such as Mg^2+^, some amino acids, thioureas, and alkaloids (quinine, strychnine, nicotine, and caffeine). The structure of the T2R receptors reflects the classic structure of 7TM receptors, characterized by possessing seven transmembrane alpha helices (Lindemann 1996; Behrens and Meyerhof 2006; Wiener et al. 2012). One of these receptors, TAS2R38, fairly strictly binds the chemical group CN=S, which is contained in many thioureas, such as phenylthiocarbamide (PTC) and 6-*n*-propylthiouracil (PROP) (Duffy et al. 2004; Kim and Drayna 2004), and is widespread in edible vegetables of the cruciferous family (Keller et al. 2002; Bell and Tepper 2006; Dinehart et al. 2006). Using a simple test, such as a strip of paper containing known concentrations of PROP placed on the front of the tongue (Bartoshuk 2000), it is possible to distinguish three groups of individuals: super tasters, who have a very high perception of bitter (threshold about 1.0 × 10^−4^ mol/L); medium tasters; and non-tasters, who receive very little or no bitter (threshold greater than 2.0 × 10^−4^ mol/L). The super-tasters are more sensitive to bitter tastes, and typically show a lower acceptance of Brassica vegetables containing molecules such as thioureas. Moreover, several studies show that super-tasters also avoid strong-tasting foods that do not contain the thiourea groups, because they also have greater perception of sweetness, spicy food, alimentary fat and alcoholic beverages (Duffy et al. 1996, 2004; Tepper and Nurse 1998; Keller et al. 2002; Dinehart et al. 2006; Hayes and Duffy 2007; Tepper 2008; Robino et al. 2014). Consequently, PROP sensitiveness could have broad implications for human health and nutrition (Bartoshuk 2000), even if not all research supports support this conclusion (Timpson et al. 2005; Drewnowski et al. 2007; Baranowski et al. 2011) Sensitivity to the bitter taste of PROP, as widely reported in the literature, has a hereditary character in that it is associated with the PAV and AVI haplotypes of the *TAS2R38* receptor gene (Kim et al. 2003; Bufe et al. 2005). The receptor gene *TAS2R38*, localized on the long arm of chromosome 7 (Drayna et al. 2003), is composed of a single exon in which there are three SNPs in gene nucleotide positions 145 (*rs713598 C/G*), *785* (*rs1726866* C/T), and 886 (rs10246939 G/A), as shown in Figure 1. The three SNPs are not synonymous and are responsible for three amino acid substitutions in the protein at positions P49A (proline/alanine), A262V (alanine/valine), and V296I (valine/isoleucine), respectively. The second and third SNPs are in perfect linkage disequilibrium (Wooding et al. 2004; Drayna et al. 2003; Kim et al. 2003; Prodi et al. 2004; Kim and Drayna 2004). The protein produced by the *TAS2R38* gene may contain the alanine–valine–isoleucine (AVI) or proline–alanine–valine (PAV) amino acid sequence. The two sequences, AVI and PAV, identify three different genotypes. Subjects with the dominant haplotype PAV/PAV express high sensitivity to the bitterness of thioureas, corresponding to the super-taster; subjects with the recessive haplotype AVI/AVI show limited or no sensitivity to thiourea, corresponding to the non-taster; and subjects with the heterozygous PAV/AVI display intermediate sensitivity, corresponding to the medium taster (Kim et al. 2003). In matters of physiology and the biochemistry of taste, the scientific community has for some time also evaluated the possibility that differences in sensitivity to the bitter taste, as well as haplotypes of the *TAS2R38* gene, may depend on CA VI protein (Padiglia et al. 2010), which is considered to have an important role in taste function. In fact, recent studies have shown that the CA VI protein acts as a trophic factor in the growth and development of the taste buds (Melis et al. 2013). The results of recent molecular studies conducted in our laboratory on individuals classified as super-tasters, medium tasters, and non-tasters showed a significant correlation between the difference in taste perception and individual polymorphism rs2274333 (A/G) located in the third exon *CA6* gene coding for CA VI (Calò et al. 2011). Specifically, it has been shown that the AA genotype and the A allele is much more frequent in individuals super-tasters, while the GG genotype and the G allele are much more frequent in non-tasters (Padiglia et al. 2010; Calò et al. 2011). It should be stressed that the studies of Padiglia et al. and Calò et al., were both conducted in a relatively homogeneous group living on the island of Sardinia. Studies among ethnically mixed populations in Brazil (Genick et al. 2011) and in the United States (Feeney and Hayes 2014) failed to find associations between PROP testing and *CA6* gene SNPs. Moreover, a recent pilot study on PROP responsiveness and thermal status was not able to confirm the association between *CA6* rs2274333 and *TAS2R38* polymorphism (Bering et al. 2014). In light of the results obtained in the course of research on the protein CA VI (Tomassini Barbarossa et al. 2011), the objective of this study was to evaluate the polymorphisms of the *TAS2R38* with our new method and its interaction with *CA6* genes in the modulation of the phenotype for sensitivity to PROP. Consequently, in this study, we took into consideration the *TAS2R38* gene haplotypes of a group of 60 subjects with variable sensitivity to PROP and preliminarily genotyped them for the rs2274333 allele (A/G) of the *CA6* gene (Tomassini Barbarossa et al. 2011). The analysis of the nucleotide sequences upstream and downstream of the SNPs showed no presence of restriction sites for any endonuclease in the three alleles of the *TAS2R38* gene (rs713598 C/G, rs1726866 C/T, and rs10246939 G/A); as such, we developed a strategy to create a restriction site artificially at each SNP for one specific enzyme. We thus used PCR-RFLP, which is considered to be a cost-effective technique for genotyping of species-specific variations. For example, it was used for the detection of the *JK* allele associated with a Kidd-null phenotype (Horn et al. 2012), for the determination of apolipoprotein E (APOE) alleles (Jiang et al. 2011) and it has been successfully utilized to identify rs1800247 SNP variant in osteocalcin in populations in China’s Guangxi Province (Liu et al. 2015).

## Methods

### Experimental strategy

The experimental strategy required that each SNP be incorporated within a PCR fragment obtained with primers capable of promoting site-directed mutagenesis in the DNA, with the purpose of subsequent use in the PCR-RFLP. The primers were designed to introduce opportune nucleotide substitutions into the amplified fragments, upstream or downstream of the SNP, in order to create a region palindromic interceptable by an endonuclease. Therefore, the SNP is one of the nucleotides present in a restriction site created artificially.

### Primer design strategy for the identification of polymorphism rs713598 C/G

**Figure 2:**
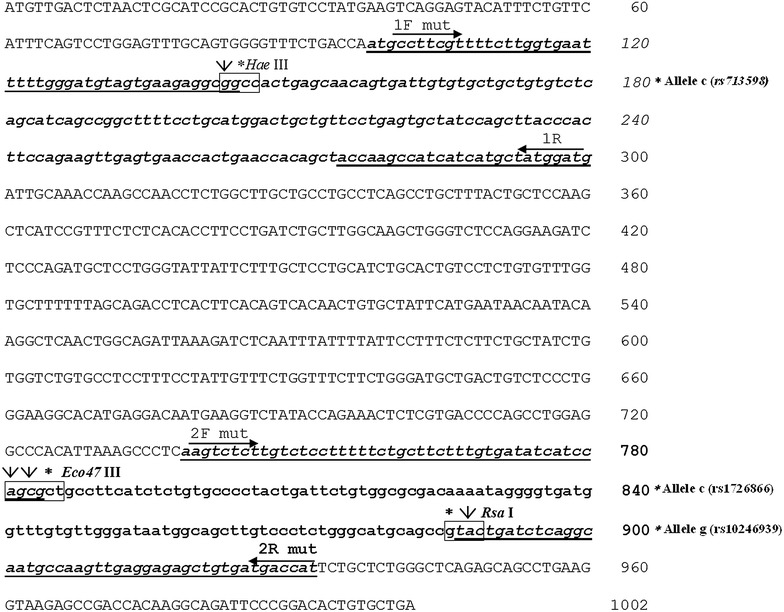
Depiction of how the mutagenesis designed in the gene results in the formation of restriction sites for the *Hae*III, *Eco47*III, and *Rsa*I enzymes when the three alleles typical of the super-taster, indicated with an* asterisk* (allele C of rs713598 C/G, allele C of rs1726866 C/T, and allele G of rs10246939 G/A), are present in the *TAS2R38* gene.

To identify polymorphism rs713598 C/G, we used a pair of primers able to amplify a fragment of 203 bp (from nucleotide 98 to nucleotide 300) of *TAS2R38.* The sequence of the *TAS2R38* gene was obtained from the NCBI gene database (accession numbers AY258597 and AY258598 for PTC non-taster and taster alleles, respectively). Figure 1 shows the full sequence and the SNPs of PTC gene. The sense primer (1Fmut 5′-atgccttcgttttcttggtgaatttttgggatgtagtgaagaggcgg-3′) used for amplification of the fragment was designed by introducing, in correspondence with the second last nucleotide, a G in place of an A, as shown in Figure 1. This mismatch is fundamental for the PCR experiments, because the A nucleotide in the sequence of the *TAS2R38* gene is replaced by a G in each of the amplification products. This creates the first G nucleotide sequence GGCC recognition for *Hae*III, thus allowing the cut of the sequence only when present in the fragment and the C allele, which is present in taster subjects, as shown in Figure 2. With the changes introduced in the fragment, it is possible to recognize the C allele from the G allele of the rs713598 SNP, because in the presence of endonuclease *Hae*III, the PCR fragment is digested into two fragments of 47 and 156 bp, respectively (Figure 2). The reverse primer (1R 5′-catccatagcatgatgatggcttggta-3′) was designed without mismatch and based on the sequence of the gene.

### Primer design strategy for the identification of polymorphisms rs1726866 C/T and rs10246939 G/A

To identify polymorphisms rs1726866 C/T and rs10246939 G/A, we designed a pair of primers that amplify a fragment of 194 bp (from nucleotide 739 to nucleotide 932) that contains both polymorphisms within its sequence (Figure 1). The sense primer (2F mut 5′-aagtctcttgtctcctttttctgcttctttgtgatatcatccagcg-3′) was designed by introducing a double mismatch in its sequence. Specifically, the second and fourth nucleotide starting from the 3′ end of the oligo contain, respectively, a C instead of a T and an A instead a T (as shown in the sequence). As illustrated above, these mismatches are essential for the PCR experiments, because the two T nucleotides in the sequence of the *TAS2R38* gene are replaced by an A and C, respectively, in each of the amplification products. This creates the first A and the third C of the recognition sequence AGCGCT for *Eco47*III endonuclease, allowing the cut of the sequence only when the fragment presents the C allele (Figure 2). The reverse primer (2R mut 5′-atggtcatcacagctctcctcaacttggcattgcctgagatcagta-3′) was designed to introduce an A instead of a C in the PCR product, leading to the formation of a restriction site, GTAC, specific to the enzyme *Rsa*I. In fact, through this modification, if the G allele typical of the super-taster is present along with the A nucleotide artificially introduced into the fragment, it leads to the formation of the first and the third nucleotide, respectively, of the cutting site recognized by the *Rsa*I endonuclease (Figure 2). In summary, by subjecting the PCR fragment to enzymatic digestion with *Eco47*III, two restriction fragments of 45 and 149 bp, respectively, are generated only if the rs1726866 SNP contains the allele C characteristic of the super-taster. If the same fragment is subjected to digestion with the *Rsa*I enzyme, the fragment is cut, even in this case, to restriction fragments of 149 and 45 bp only if the rs10246939 SNP presents the G allele characteristic of the super-taster. All primers used in PCR experiments were synthetized by Invitrogen 50 nmol scale, desalted (Europrim-Invitrogen, Cambridge, UK).

### Subjects

A total of 60 healthy subjects, all non-smokers, who were already genotyped for the *CA6* rs2274333 SNP (Padiglia et al. 2010; Tomassini Barbarossa et al. 2011) were recruited for this research. Their mean age was 25 years, ranging 20–29 years. All of the subjects had measurable thresholds for common chemosensory stimuli and were free of medications that might affect taste or odor perception. The 60 participants were classified for PROP bitter taste status as super-tasters, medium tasters, or non-tasters using standard procedures (Tepper et al. 2001, Rankin et al. 2004). The PROP phenotype of each subject was assessed by a scaling method for taster status classification and by detection threshold measurement. The classification of subjects by taster status was determined by their PROP and NaCl ratings, using the 3-solution test. Each group had an equal number of super-tasters:medium tasters:non-tasters ratio (20:20:20). The participants were informed about the procedure and the objective of the work, and each subject reviewed and signed an informed consent form at the beginning of the protocol. The study was approved by the Ethical Committee of the University Hospital of Cagliari, Italy.

### Preparation of template DNA and PCR conditions

Genomic DNA was extracted from saliva samples using the Invitrogen Charge Switch Forensic DNA Purification kit (Invitrogen, Carlsbad, CA, USA) according to the manufacturer’s protocol. The concentration was estimated by measurements of OD260. Purified DNA samples were stored at or below −20°C until use. The PCR reaction was carried out in a total volume of 25 μL and contained 250 ng DNA, 10 pmol of each primer, 1.5 mM MgCl_2_, 100 mM Tris–HCl, pH 8.3, 50 mM KCl, 200 μM of dNTP mix, and 1.5 U of Hot Master Taq Eppendorf. Reactions were performed in a Personal Eppendorf Mastercycler (Eppendorf, Hamburg, Germany). Using pairs of 1F mut/1R primers specific for the analysis of 1° SNP (C/G) and 2F mut/2R mut specific for the analysis of the 2° and 3° SNP (C/T and G/A), we obtained amplification products of 203 and 194 bp, respectively, whose dimensions reflected those of the expected products. Amplification conditions for the 1Fmut/1R fragment consisted of initial denaturation at 95°C for 2 m, followed by 35 cycles of denaturation at 95°C for 30 s, annealing at 63°C for 30 s, and extension at 72°C for 30 s. Amplification conditions for the 2Fmut/2R fragment consisted of initial denaturation at 95°C for 2 m, followed by 35 cycles of denaturation at 95°C for 45 s, annealing at 68°C for 30 s, and extension at 72°C for 30 s. A final extension was conducted at 72°C for 5 m for both amplification reactions. Negative controls (water instead of human DNA) were run with every PCR, and standard precautions were taken to avoid contamination.

### RFLP analysis for genotyping

**Figure 3:**
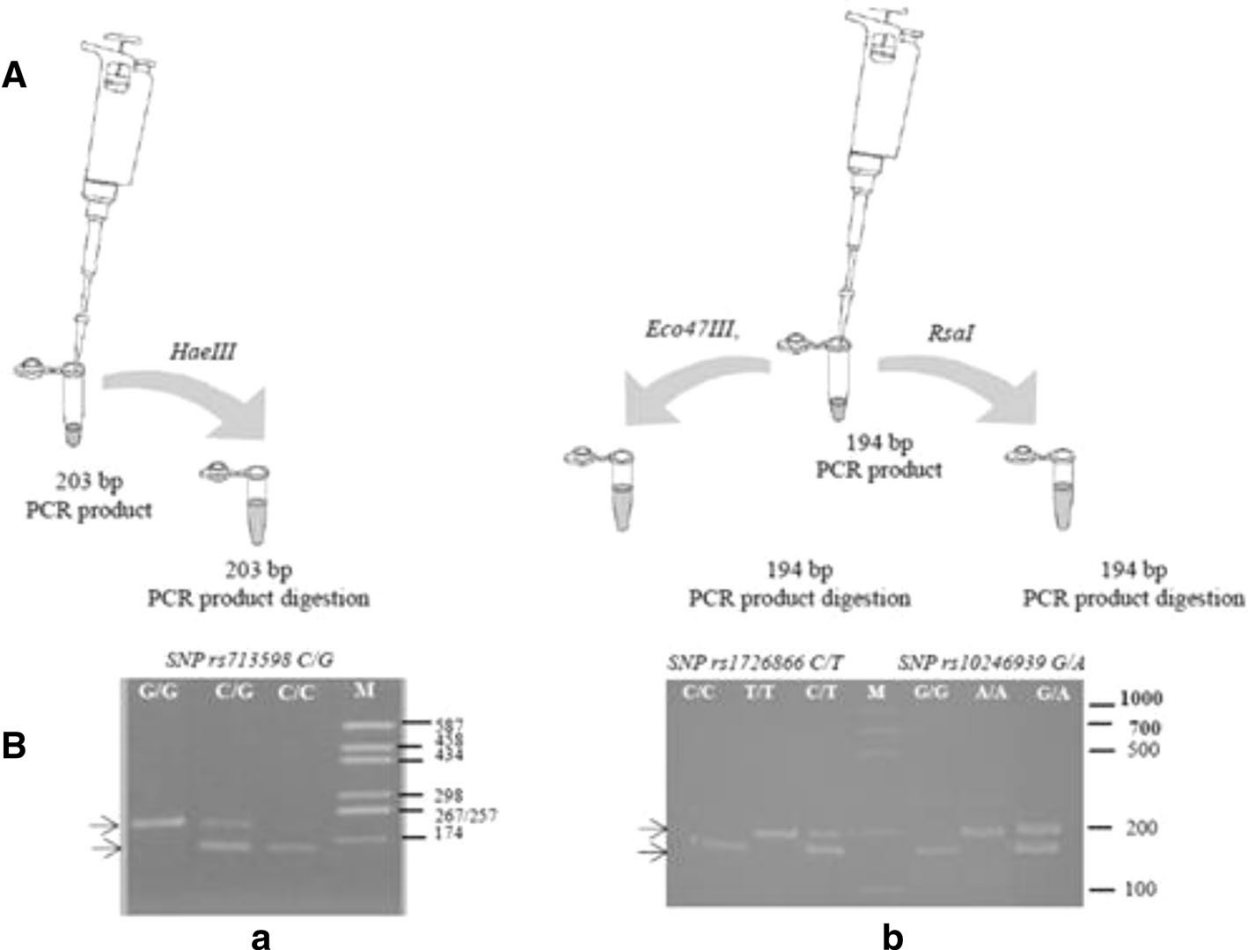
Assay design (**A**) and example of the results (**B**) obtained with polymerase chain reaction (PCR)-restriction fragment length polymorphism method. A 203 bp fragment *TasR38* gene that covers the first SNP site of interest was amplified and subsequently digested with *Hae*III restriction enzyme that recognizes only the C allele (*a*). A 194 bp fragment *TasR38* gene that covers the second and third SNP sites of interest was amplified and subsequently digested with *Eco47*III restriction enzyme that recognizes only the C allele and with *Rsa*I that recognizes the G allele. The resulting fragments that show the genotype of each individual were analyzed on polyacrylamide gel in parallel with the untreated PCR products. *a* M, size marker; G/G, C/G, and C/C correspond to the SNP alleles of polymorphism rs713598. The *arrows* indicate the DNA fragments (203, 156 bp) obtained after *Hae*III restriction enzyme digestion. A 47 bp restriction fragment was eluted from the gel. *b* M, size marker; on the *left* of the size marker, C/C, T/T, and C/T correspond to the SNP alleles of polymorphism rs1726866; on the *right* of the size marker, G/G, G/A, and A/A correspond to the SNP alleles of polymorphism rs10246939. The *arrows* indicate the DNA fragments (194, 149 bp) obtained after *Eco47*III and *Rsa*I restriction enzyme digestion. A 45 bp restriction fragment was eluted from the gel. The gel was stained with ethidium bromide.

A 5 μL aliquot of the PCR reaction fragment of 203 bp containing the rs713598 G/C SNP was mixed with a 15 μL solution containing 2 μL 10× NE buffer (50 mM NaCl, 10 mM Tris-HCl, 10 mM MgCl_2_, 1 mM dithiothreitol, pH 7.9), 0.2 μL *Hae*III (10,000 U/mL) (Sigma-Aldrich, St. Louis, MO, USA), and 11 μL sterile deionized H_2_O, then incubated at 37°C for 2 h (Figure 3). To identify SNPs rs1726866 C/T and rs10246939 G/A, we subjected the 194 bp fragment 2Fmut/2Rmut at two different enzymatic digestions in the presence endonuclease *Eco47*III and *Rsa*I, respectively. A fragment modified in that manner shows the sequence AGCGCT for *Eco47*III and the sequence GTAC for *Rsa*I. A 5 μL aliquot of the 194 bp PCR reaction fragment of was then mixed with a 15 μL solution containing 2 μL 10× buffer O (50 mM Tris-HCl, 10 mM MgCl_2_, 100 mM NaCl, and 0.1 mg/mL BSA pH 7.5), 2 μL *Eco47*III (10 U/mL) (ThermoFisher Scientific, Waltham, MA, USA), and 11 μL sterile deionized H_2_O. Similarly, a 5 μL aliquot of the same PCR reaction fragment was mixed with a 15 μL solution containing 2 μL 10× Tango buffer (33 mM Tris-acetate, 10 mM Mg-acetate, 66 mM K-acetate, and 0.1 mg/mL BSA; pH 7.9), 2 μL *Rsa*I (10 U/mL) (ThermoFisher Scientific), and 11 μL sterile deionized H_2_O. Each reaction (*Eco47*III and *Rsa*I) reaction mixture was mixed and then incubated at 37°C for 2 h (Figure 3). The digest (10 μL) was mixed with 3 μL of loading buffer and electrophoresed on a 10% vertical polyacrylamide gel. Silver nitrate and ethidium bromide staining were carried out according to the methods of Herring et al. (1982) and Sambrook et al. (1989), respectively. The digested pUC18 DNA *Hae*III plasmid (Sigma-Aldrich) and Edu-PCR MW Ruller (Bio-Rad Laboratories, Inc. Hercules, California, USA) were used as an MW marker. All PCRs and digestions were conducted in triplicate for each DNA sample. Figure 3 shows an example of the electrophoretic profiles obtained in the presence of the three SNPs of the *TasR38* gene. In order to verify the accuracy of the PCR-restriction fragment length polymorphism method, all PCR samples were sequenced by the Sanger method with an ABI Prism 310 automated sequencer and for more accuracy and better confirmation of results, the genotyping was based on both the forward and reverse strands. Translations of nucleotide sequences were performed using ExPASy translate routine software (http://ca.expasy.org/). Allelic frequencies were determined and statistical analysis was performed using the Chi square (*χ*^2^) test. Fisher’s method (Genepop software version 4.0; http://kimura.univ-montp2fr/~rousset/Genepop.htm) was used to test *TAS2R38* genotype distribution and haplotype frequencies, as well as genotype distribution and allele frequencies of the *CA6* gene polymorphism rs2274333 (A/G) in terms of PROP status. Genetic differences among the three taster groups based on the distribution of the *TAS2R38* and *CA6* genotype combinations were tested by the Markov Chain method (Arlequin software version 3.1; http://cmpg.unibe.ch/software/arlequin3).

## Results and discussion

In this study, using mutated primers, we developed a PCR-RFLP method for the analysis of rs713598 C/G, rs1726866 C/T and rs10246939 G/A *TAS2R38* SNPs. The molecular characterization of the *TAS2R38* alleles was conducted after creating artificial restriction sites upstream or downstream of the SNPs of interest, as none of the three polymorphisms contributes to the formation of a restriction site for a specific endonuclease. To identify the first SNP (rs713598 C/G), the DNA was amplified with a mutated sense primer that creates, in PCR products, the first G nucleotide sequence GGCC recognition for *Hae*III, thus allowing the cut of the sequence only when present in the fragment and the C allele, characteristic of taster subjects (Figure 2). In order to identify the second and third SNPs (rs1726866 C/T and rs10246939 G/A), we performed only one PCR reaction with both mutated pair primes. The sense primer was designed by introducing a double mismatch in its sequence, with the purpose of creating the first A and the third C of the recognition sequence AGCGCT for *Eco47*III endonuclease, allowing the cut of the sequence only when the fragment presents the C allele typical of the taster. The reverse primer was designed to introduce, in the same PCR product, the A nucleotide of the restriction site, GTAC, recognized by the *Rsa*I enzyme. Through this modification, if the G allele typical of the taster is present along with the A nucleotide artificially introduced into the fragment, it leads to the formation of the first and the third nucleotides, respectively, of the cutting site recognized by the *Rsa*I endonuclease (Figure 2). In order to verify the accuracy of the enzymatic digestions, all PCR samples were sequenced on both the forward and reverse strands with an ABI Prism automated sequencer. As has been widely reported in the literature, we found a match between PROP taster status and *TAS2R38* gene haplotypes in the individuals studied. The statistical methods (Fisher’s method) showed, as fully described in the literature, a significant correlation (p < 0.0001), showing that the PAV haplotype is widely distributed in the general PROP tasters, while the AVI haplotype was distributed in non-tasters. As shown in Additional file 1: Table S1, and as was further observed by Calò et al. (2011), the large proportion of heterozygous PAV/AVI in the super-taster phenotype suggests that the *TAS2R38* gene haplotypes justified the sensitivity to the bitter taste of PROP only in part, distinguishing only two phenotypes for the sensitivity to PROP: tasters and non-tasters. Molecular analysis of the rs2274333 (A/G) polymorphism of the *CA6* gene, conducted in our previous study on the same 60 subjects, showed that the A allele and the AA genotype are more prevalent in the super-taster phenotype, while the GG genotype and the G allele are more prevalent in the non-taster phenotype (Tomassini Barbarossa et al. 2011). In view of the results obtained in the present study, we correlated the *TAS2R38* gene polymorphisms with the rs2274333 (A/G) SNP of the *CA6* gene in the three groups of subjects. According to the results obtained by Calò et al. (2011), a high percentage of PAV/AVI subjects, observed after restriction analysis and validated by sequencing data, can justify a super-taster phenotype when this heterozygous status is associated with the A allele of the rs2274333 *CA6* polymorphism (Additional file 2: Table S2). Thus, the high sensitivity to PROP in the Sardinian population super-tasters seems to be determined by the association (*p* value <0.0001, calculated using the Markov Chain method) of gene haplotype PAV *TAS2R38* with at least one A allele of the *CA6* gene.

## Conclusions

Even if the mutation does not result in a restriction site difference, it was possible to exploit the difference between the two allelic forms of each SNP of *TAS2R38* gene, by amplification-created restriction site PCR. Thus, by using the PCR-RFLP technique developed in our laboratory, we established a simple, efficient and low-cost method that can be used in any laboratory equipped with basic instrumentation for the study of DNA, to determine the allelic forms of the *TAS2R38* gene. Some of the important advantages of the PCR-RFLP technique include inexpensiveness and the lack of need for advanced instrumentation or extensive training of laboratory staff. Not all research laboratories have a DNA sequencer or else a real-time instrument to observe the SNPs by labeled primers or expensive TaqMan-type probes. Thus, such laboratories must send their samples to outside laboratories that are equipped with specific instrumentation. Some of the disadvantages of the PCR-RFLP technique could be that this experimental approach requires relatively large amounts of hands-on time when compared to other techniques such as direct sequencing. With regard to genotyping by sequencing, to obtain the nucleotide sequence it is, however, obligatory to have subjected the DNA to a PCR reaction. Also, the time employed for digestion of the PCR products using the PCR-RFLP method may also be the same as the time required for sequencing, especially if the rapid digestion restriction enzymes are used. Thus, the method described above, validated by direct sequencing, could be useful to reconstruct *TAS2R38* gene haplotypes, and might represent an experimental strategy to identify differences in gustatory sensibility. Since taste perception plays a key role in determining individual food preferences and dietary habits, the method could be applied as a test of nutrigenetics in studies aimed at understanding eating behaviors. Until recently, *TAS2R38* has been considered the only gene involved in gustatory sensibility to the bitter taste, but the data obtained in this study confirm, in a Sardinian genetic isolate, that *TAS2R38* haplotypes and the polymorphisms rs2274333 (A/G) of the carbonic anhydrase VI gene might cooperate in the modulation of the phenotype related to sensitivity to PROP. Genetic differences in genes involved in taste perception between ethnic groups may contribute to differences in patterns of diet. Understanding these differences in genetic variations may help to explain ethnic differences in the risk for chronic diseases, and could lead to the development of appropriate public health measures of prevention.

## Additional files

Additional file 1: Table S1. Distribution of genotypes and frequency of haplotypes TAS2R38 in agreement with the PROP taster status. Additional file 2: Table S2. Distribution of the combinations of genotypes of the TAS2R38 and CA6 genes according to PROP taster status.
