# Supplementary material for: Development of a molecular method for the rapid screening and identification of the three functionally relevant polymorphisms in the human TAS2R38 receptor gene in studies of sensitivity to the bitter taste of PROP
Source: Springerplus. 2015 Jun 9;4:246. doi: 10.1186/s40064-015-1045-0 (PMC4467798; doi:10.1186/s40064-015-1045-0)
Supplement: Additional file 1: Table S1. — Distribution of genotypes and frequency of haplotypes TAS2R38 in agreement with the PROP taster status. [file 40064_2015_1045_MOESM1_ESM.pdf]

Table 1 Distribution of genotypes and frequency of haplotypes *TAS2R38* in agreement with the PROP taster status

	PROP status						p-value [a]
	Super-taster		Medium-taster		Non-taster		
	n	%	n	%	n	%	
**Genotype**							
PAV/PAV	9	45.00	2	10.00	0	0	< 0.0001
AVI/AVI	0	0	1	5.00	19	95.00	
PAV/AVI	11	55.00	17	85.00	1	5.00	
**Haplotype**							
PAV	29	72.50	21	52.50	1	2.50	< 0.0001
AVI	11	27.50	19	47.50	39	97.50	

[a] p-value derived from Fisher's method (n= 60)
