# Supplementary material for: Development of a molecular method for the rapid screening and identification of the three functionally relevant polymorphisms in the human TAS2R38 receptor gene in studies of sensitivity to the bitter taste of PROP
Source: Springerplus. 2015 Jun 9;4:246. doi: 10.1186/s40064-015-1045-0 (PMC4467798; doi:10.1186/s40064-015-1045-0)
Supplement: Additional file 2: Table S2. — Distribution of the combinations of genotypes of the TAS2R38 and CA6 genes according to PROP taster status. [file 40064_2015_1045_MOESM2_ESM.pdf]

Table 2 Distribution of the combinations of genotypes of the *TAS2R38* and *CA6* genes according to PROP taster status

Genotype *TAS2R38* *CA6*	PROP status						p-value [a]
	Super-taster		Medium-taster		Non-taster		
	n	%	n	%	n	%	
PAV/PAV-AA	8	40.00	1	5.00	0	0	< 0.0001
PAV/PAV-AG	1	5.00	0	0	0	0	
PAV/PAV-GG	0	0	1	5.00	0	0	
AVI/AVI-AA	0	0	1	5.00	6	30.00	
AVI/AVI-AG	0	0	0	0	1	5.00	
AVI/AVI-GG	0	0	0	0	12	60.00	
PAV/AVI-AA	9	45.00	9	45.00	0	0	
PAV/AVI-AG	2	10.00	7	35.00	0	0	
PAV/AVI-GG	0	0	1	5.00	1	5.00	

[a] p-value derived from Markov chain method (n = 60).

[a] **p-value derived from Fisher's method (n= 60)**

< 0.0001
